# Provider Antibody Serology Study of Virus in the Emergency Room (PASSOVER) Study: Special Population COVID-19 Seroprevalence

**DOI:** 10.5811/westjem.2021.1.50058

**Published:** 2021-04-09

**Authors:** Theodore W. Heyming, Terence Sanger, Aprille Tongol, John Schomberg, Kellie Bacon, Bryan Lara

**Affiliations:** * Children’s Hospital of Orange County, Department of Emergency Medicine, Orange, California; † University of California, Irvine, Department of Electrical Engineering and Computer Science, Irvine, California; ‡ University of California, Irvine, Department of Emergency Medicine, Irvine, California; § Children’s Hospital of Orange County, Research Institute, Orange, California; ¶ Children’s Hospital of Orange County, Department of Nursing, Orange, California

## Abstract

**Introduction:**

Limited data on the seroprevalence of severe acute respiratory syndrome coronavirus 2 (SARS-CoV-2) among healthcare workers (HCW) are publicly available. In this study we sought to determine the seroprevalence of SARS-CoV-2 in a population of HCWs in a pediatric emergency department (ED).

**Methods:**

We conducted this observational cohort study from April 14–May 13, 2020 in a pediatric ED in Orange County, CA. Asymptomatic HCW ≥18 years of age were included in the study. Blood samples were obtained by fingerstick at the start of each shift. The inter-sampling interval was ≤96 hours. The primary outcome was positive seroprevalence of SARS-CoV-2 as determined with an antibody fast detection kit (Colloidal Gold, Superbio, Timisoara, Romania) for the SARS-CoV-2 immunoglobulin M/immunoglobulin G (IgM/IgG) antibody.

**Results:**

A total of 143 HCWs participated in the study. Overall SARS-CoV-2 seroprevalence was 10.5% (n = 15). Positive seroprevalence was classified as IgG only (4.9%), IgM+IgG (3.5%), or IgM only (2.1%). SARS-CoV-2 was detected by reverse transcription polymerase chain reaction RT-PCR in 0.7% of the overall study population (n = 1). Samples obtained on Day 1 indicated seropositivity in 4.2% of the study population (n = 6). Subsequent seroconversion occurred in 6.3% of participants (n = 9). The rate of seroconversion was linear with a rate of approximately one new case every two days, starting at Day 9 of the study.

**Conclusion:**

We observed a linear rate of seroconversion to SARS-CoV-2–positive status among asymptomatic HCWs who underwent daily symptom surveys and temperature screens in an environment with universal source control. Rapid antibody testing may be useful for screening for SARS-CoV-2 seropositivity in high-risk populations, such as HCWs in the ED.

## INTRODUCTION

The healthcare system plays a pivotal role in minimizing disease transmission, protecting healthcare personnel, and preserving health services during the current pandemic. By February 11, 2020, 3019 healthcare workers (HCW) in China had contracted coronavirus disease 2019 (COVID-19). By April 24, 2020, 19,942 HCWs in Italy had contracted the disease.^1^,^2^ Guidelines for infection control released by the US Centers for Disease Control and Prevention (CDC) include universal source control for everyone entering a healthcare facility, regardless of symptoms, through mandatory mask usage, and active screening of all personnel, patients, and visitors for fever and symptoms of COVID-19 before entry.^3^ Similar recommendations have been made outside the US^4^

Although data on the infection rates of HCWs in the US are limited, preliminary data from California suggest that HCWs represent an alarming 7.7% of all known COVID-19 cases.^5^ In a survey of 13 academic medical centers that included 3248 HCWs, 6% were seropositive for severe acute respiratory syndrome coronavirus 2 (SARS-CoV-2). Among those who tested positive, 29% were asymptomatic, and 69% had not previously known that they were infected with SARS-CoV-2.^6^ An earlier detailed analysis demonstrated that only 18% of HCW who tested positive were known to have been infected in a healthcare setting, suggesting that HCWs may be more likely to contract the disease outside of the healthcare setting.^7^ As of 3/26/2021, there were 452,706 cases of COVID-19 among healthcare personnel, and 1,505 deaths from COVID-19.^8^

The current practice for diagnosing COVID-19 is based on the use of reverse transcriptase polymerase chain reaction (RT-PCR) to test for the presence of SARS-CoV-2 in pharyngeal or respiratory specimens. Current epidemiologic data are based on samples from symptomatic patients at high epidemiologic risk and are likely to underestimate the true prevalence of infection. Because many infections are subclinical, serologic methods can play an important role in determining the true prevalence of COVID-19.^9^ Early serologic studies have reported high sensitivity in detecting SARS-CoV-2 infection, with antibodies to virus detected 6–15 days after disease onset.^10^ Unlike RT-PCR positivity, SARS-CoV-2 antibodies persist for at least six weeks and remain detectable throughout the course of disease.^11^,^12^ Multiple serologic tests for COVID-19 have been developed, including a recently approved lateral flow assay. However, there is concern over the limitations of these tests, such as cross-reactivity with antibodies to other human coronaviruses. Such tests typically detect antibodies against only one or two antigens. Nonetheless, serology testing may help to characterize the rate of spread of COVID-19 within healthcare settings.

In this study we sought to determine the seroprevalence of SARS-CoV-2 antibodies and to calculate the rate of seroconversion in a population of HCWs within a pediatric emergency department (ED) in Orange County, CA.

## METHODS

### Study design

Staff members in the Julia and George Argyros Emergency Department at CHOC Children’s Hospital participated in the study during each shift from April 14–May 13, 2020. The study was approved by the CHOC Children’s Institutional Review Board. Signed informed consent was obtained from all study participants. The final enrollment number represents those participants who voluntarily consented; there were no exclusions. All study participants were ≥18 years of age and active employees in the CHOC Children’s ED with direct patient contact, or clerical staff present in the same area as patients. This included physicians, physician assistants, nurse practitioners, nurses, medical technicians, secretaries, monitor technicians, and additional administrative staff. All subjects were asymptomatic and afebrile, as all employees underwent daily pre-shift active verbal screening for symptoms and/or household exposure, as well as daily temperature measurements with a Masimo TIR-1 noncontact clinical-grade infrared thermometer (Masimo Corporation, Irvine, CA) prior to entering the hospital. Personnel with positive screening results were barred from entering the hospital. Any exposure of HCWs to patients or other HCWs infected with SARS-CoV-2 was traced according to CDC guidelines. The associated rate of infection was 0.28% (1 out of 362 exposures).^13^ Funding for this study was provided by CHOC Children’s Hospital.

**Population Health Research Capsule**

What do we already know about this issue?

*During the current pandemic, publicly available data on the seroprevalence and seroconversion of SARS-CoV-2 among healthcare workers has been limited.*

What was the research question?

*This study measured the seroprevalence of SARS-CoV-2 in a population of pediatric emergency department health-care workers.*

What was the major finding of the study?

*We observed a linear rate of seroconversion to SARS-CoV-2–positive status in asymptomatic healthcare workers.*

How does this improve population health?

*Rapid antibody testing may be useful for screening for SARS-CoV-2 seropositivity in high-risk populations, such as healthcare workers in the emergency department.*

### Serologic testing

Blood samples were obtained every 96 hours or upon arrival to the HCW’s shift after the 96 hours, until the end of the 30-day study period. All samples were tested with the COVID-19 Rapid Test Kit IgG + IgM (Colloidal Gold) (Superbio Biomedical Company, Rancho Cordova, CA). At the time of the study, the Superbio test was approved by the US Food & Drug Administration (FDA) under an umbrella emergency use authorization (EUA) for SARS-CoV-2 antibody tests.^14^

Through combined analysis of three possible positive results (immunoglobulin M (IgM) only, immunoglobulin G (IgG) only, IgM+IgG), the Superbio kit has overall sensitivity of 100% and specificity of 83.8%.^15^ The manufacturer reported that this kit accurately identified 70 nCoV-2019 virus nucleic acid-positive blood samples and 70 negative blood samples. The kit also yielded accurate results when tested on 135 negative blood samples. These values correspond to similar results from studies of other lateral flow assays, with reported sensitivity ranging from 65–93% and specificity ranging from 97.2–99.8%.^16^

Fingerstick sampling and antibody testing were performed by trained and certified ED personnel. Consensus between two investigators was needed to declare a positive result. Upon a positive result for either IgM or IgG, a new fingerstick sample was obtained, and the testing procedure was repeated. The daily test result was considered positive only when the results were concordant between test and retest. Based on previous reports that a two-step screening approach helps to identify early-stage disease in at-risk populations,^17^ all seropositive participants underwent confirmatory serum antibody testing with the Abbott Architect SARS-CoV-2 IgG assay (Abbott Laboratories Inc., Chicago, IL) within one month of their first positive antibody test.

### Collection of Nasopharyngeal Swab Specimens

A nasopharyngeal swab (NPS) specimen was collected from each participant on the date of study entry. The NPS specimens were collected by trained healthcare professionals in accordance with CDC recommend-ations.^18^ Samples were placed in a viral transport medium liquid supplied to us by the vendor laboratory. Specimens were kept at 2–8°C for up to 72 hours (hr), and then transported at −70°C for RT-PCR–based detection of SARS-CoV-2 (BioReference Laboratories, Elmwood Park, NJ). All assays performed at BioReference Laboratories have been validated and approved under the US FDA Emergency Use Authorization (EUA) for diagnostic testing.

### Reverse Transcriptase-Polymerase Chain Reaction

In cases where a healthcare worker had newly tested IgM positive, the following procedures were performed: repeat RT-PCR (if no previous RT-PCR had been obtained ≤72 hr) and expanded multiplex PCR (Biofire FilmArray, BioFire Diagnostics LLC, Salt Lake City, UT) for an additional 14 viruses, including coronaviruses associated with the common cold (229E, HKU1, NL63, OC43). Participants who newly tested IgG positive underwent repeat RT-PCR if no previous RT-PCR had been obtained within ≤72 hr.

### Data Analysis

**Figure 1:**
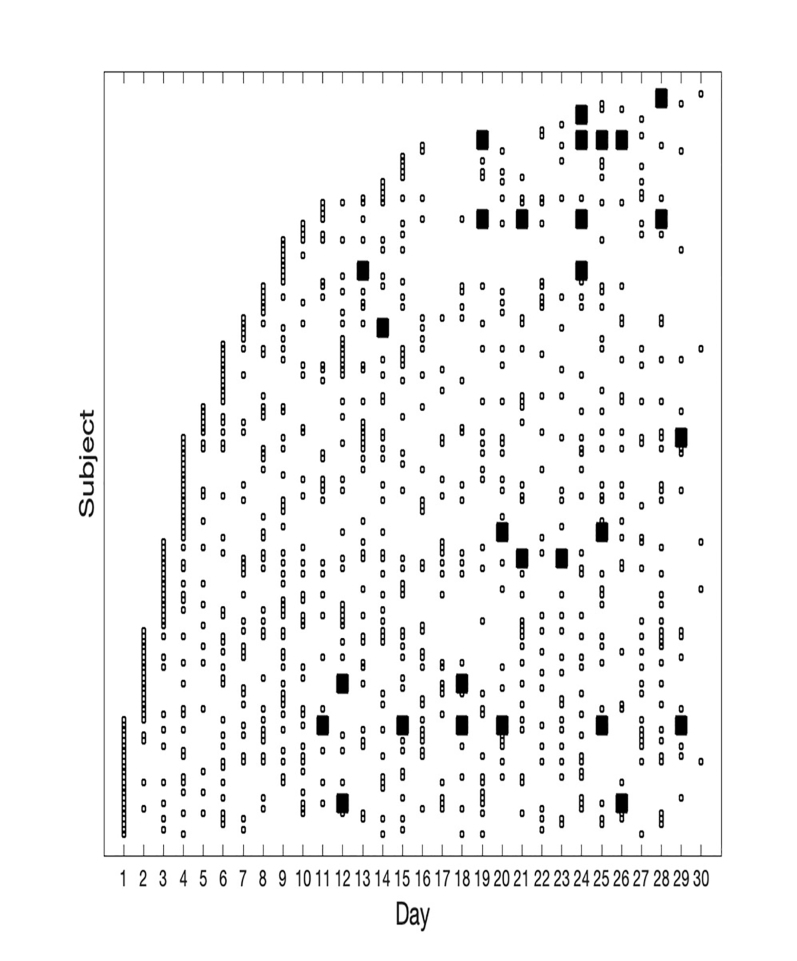
Daily tests and IgG positivity (via Antibody Fast Detection Kit (Colloidal Gold) Superbio, Timisoara, Romania) by subject, ordered by date of subject entry. Vertical axis: subjects ordered from earliest entry (bottom) to latest entry (top). Horizontal axis: day of the study. Small squares: negative IgG test. Large squares: positive IgG test.

**Figure 2:**
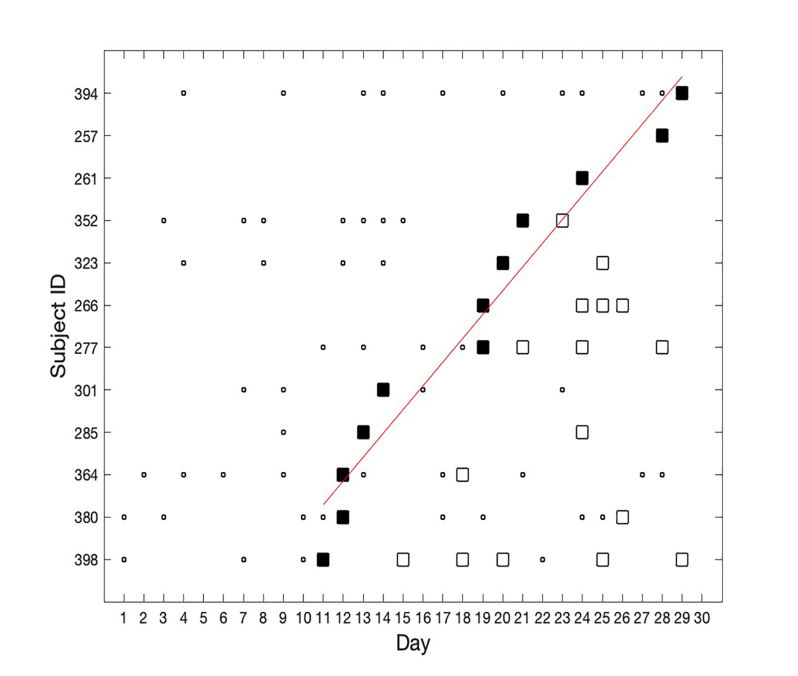
Subjects with IgG positivity (via Antibody Fast Detection Kit (Colloidal Gold) Superbio, Timisoara, Romania), ordered by the first day on which each subject became positive. Vertical axis: subject identification number ordered from earliest day of IgG seropositivity (bottom) to latest day (top). Horizontal axis: day of the study. Small squares: negative IgG test. Large solid squares: first positive IgG test. Hollow squares: subsequent positive IgG test. Red line: linear regression of initial IgG positivity by day of study with a slope of 0.56 cases per day. *ED*, emergency department; *PA*, physcian assistant; *NP*, nurse practitioner

Subjects were sorted by the day of study entry to demonstrate total enrollment and the overall pattern of conversion to seropositivity (Figure 1). The seropositive subgroup was sorted by the day of conversion to seropositivity to show the rate of acquisition in the cohort (Figure 2). Least-squares linear regression of the number of seropositive subjects by day of seroconversion was calculated and plotted to determine whether the rate of seroconversion was approximately linear over time.

## RESULTS

The study ultimately enrolled 143 of 200 ED personnel, for a total participation rate of 72.5% (143/200). Among 143 participants, physicians accounted for 12% (n = 17), allied health professionals for 8% (n = 11), registered nurses for 41% (n = 58), ED technicians for 73% (n = 21), unit secretaries for 3% (n = 4), and administrators for 54% (n = 7). The only subject who withdrew from the study had no symptoms during the study period, and all testing for this individual was negative. The table presents the demographics for the study population.

At the time of study entry, 35% of the study cohort had known exposure to a COVID-19-positive individual (including either a household or work contact) within the preceding five days. The results of participant surveys indicated that no participant who tested positive for SARS-CoV-2 antibodies had known exposure within the five days preceding seroconversion. Reviews of shift schedules, participant interviews, door entry logs, and the electronic health record provided no evidence of contact with individuals either suspected or known to have COVID-19, either within the workplace (coworkers and patients) or outside the workplace, within an 11-day period prior to any participant’s first positive result. No situations of increased risk for exposure to SARS-CoV-2 were identified. However, the possibility that ED HCWs were exposed to SARS-CoV-2 cannot definitively be ruled out.

**Figure 3:**
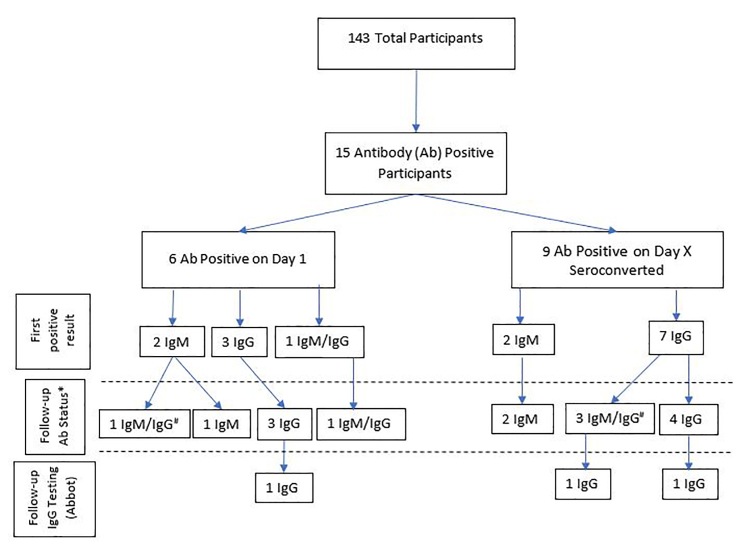
Flow diagram of results for antibody positive participants in study of seroprevalence of COVID-19 in a pediatric emergency department. *IgM*, immunoglobulin M; *IgG*, immunoglobulin G.

The study group (n = 143) yielded 896 antibody test results, including 40 values from 15 study participants who were IgM positive only, IgG positive only, or IgM+IgG positive. Figure 3 displays a flow chart of study participants found to be positive, with a breakdown of positive result categories.

To determine test-retest reliability, a second test was performed within five minutes of obtaining a positive value. The 40 positive tests included 30 IgM-positive results and 32 IgG-positive results. Twenty-eight out of 30 IgM tests were retested. In two cases, the study participants refused to undergo repeat testing. The second test yielded a positive result in only 15/28 cases (53.5%). Thirty out of 32 IgG tests were retested, and the second test remained positive in 23/30 (76.6%). For the 15 patients with two consecutive antibody-positive results, follow-up IgG testing was performed with the Abbott Architect assay within 4 weeks of obtaining the first positive Superbio test result. Three of 15 antibody-positive participants (20%) also tested positive for IgG using the Architect assay. All three of these participants had also tested positive for IgG on the Superbio test (Figure 3).

Remarkably, only one study participant received a positive RT-PCR result 1/143 (0.7% of participants). This participant also tested negative for antibodies on the same day the PCR specimen was obtained. This participant did not complete additional antibody testing. Negative results were obtained for all first-time RT-PCR tests of the 15 seropositive individuals, including the six participants who tested positive on Day 1, as well as the nine participants who seroconverted during the study period. Because of the seroconversion observed in a portion of the study population, five additional follow-up RT-PCR tests were completed on the 15 seropositive individuals. All follow-up RT-PCR tests were negative.

The study protocol included performance of a respiratory multiplex panel for participants with IgM-positive status. The study protocol also required participants to repeat the respiratory panel if they had fluctuating IgM results. Ultimately, nine expanded respiratory panel multiplex PCR tests were completed on eight participants who tested positive for IgM. One IgM-positive participant refused testing. All nine tests were negative for the four common coronavirus species. One of the nine tests was positive for both rhinovirus and enterovirus.

Figure 1 shows the results obtained for IgG seropositivity. The first seropositive case was identified on Day 11. Figure 2 shows the results for the IgG-positive subjects. The rate of seroconversion was approximately linear, at a rate of 0.56 seroconversions per day, from Days 11–30. R-squared fit to the linear model was 0.95 (Matlab Statistics Toolbox, Natick, MA). Several subjects had negative serology tests subsequent to their initial positive test.

## DISCUSSION

The aim of this study was to determine SARS-CoV-2 seroprevalence and the rate of seroconversion in HCWs in a pediatric ED in California through frequent testing over a one-month period. Orange County, which borders Los Angeles County, has not obtained large-scale seroprevalence data. After adjusting for the population of working-age individuals (n = 1,752,199), the known population prevalence of COVID-19 (as determined by RT-PCR) was 0.05% at the start of the study period. This figure increased to 0.17% by the end of the study period.^19^

The acquisition of seropositivity in our study group appeared to follow a linear trend, which is not consistent with the exponential rate of growth that would be expected for transmission within a closely interacting group of people. This finding may suggest that most infections were transmitted through community exposure, rather than via co-workers; however, our sample size is too small to draw a definitive conclusion. Notably, no subject became symptomatic during the study.

Previous studies have indicated that SARS-CoV-2 IgM may be used as an acute-phase marker for recent infection.^20^,^21^ However, there are multiple reports on various viruses, including SARS coronavirus, that suggest that IgM antibodies against viral proteins can persist for months after an acute infection.^22^–^25^ In our study, two participants tested IgM positive on Day 1, and another two participants tested IgM positive as the first marker of seroconversion. Care must be used when interpreting seropositivity for IgM as evidence of acute exposure or infectivity status and in determining the validity of the results obtained for these four participants.

Seroprevalence data are important to understand the scale and spread of the pandemic.^26^ The seroprevalence of SARS-CoV-2 IgM/IgG in our cohort of HCWs was 10.5% (15/143). The seroprevalence of SARS-CoV-2 IgG was 8.4% (12/143). These values are higher than the range of seropositivity reported by Los Angeles County (2.8–5.6%) for the general population 40 miles from our study site during the study period. Studies of two other general populations in the US reported seroprevalences of 1.5% and 1.79%. Furthermore, seroprevalence estimates may be up to 55-fold higher than estimates based on the results of RT-PCR.^16^,^27^ One study conducted in Germany reported that the overall Sars-CoV-2 IgG seroprevalence was lower in HCWs in an adult acute hospital setting (1.6%), compared with other high-risk groups (5.4%).^28^

Discrepancies between the seropositivity prevalence reported in this study and the values reported by others may reflect methodological differences between studies. One factor may have been the quality of the antibody tests used for serological testing. The current pandemic has severely limited the available supply of antibody test kits. The Superbio antibody test kit, which has overall sensitivity of 100% and specificity of 83.8%,^15^ was available for use at the time of the study, and its stated sensitivity and specificity are within the range of reported values for many other test kits.

We increased the reliability of our results by using a two-step algorithm for confirmation: with the lateral-flow antibody test (Superbio) served as the initial screening test, and the Architect assay (Abbott) served as the confirmatory test. This two-step approach has been used previously as a highly sensitive and specific noninvasive tool for the detection of seropositivity.^17^ Notably, the Architect assay has received EUA for the detection of SARS-CoV-2 from the US Food and Drug Administration (FDA).^29^ The manufacturer’s instructions state that the Architect assay, when used to analyze serum blood samples has SARS-CoV-2 IgG specificity of 99.9%.^17^ Follow-up IgG antibody testing with the Abbott assay was completed on all 15 participants found to be antibody positive with the Superbio assay. Only 3/15 (20%) of our antibody-positive participants were IgG-positive on this follow-up test.

Now that a year has passed since the onset of the current COVID-19 pandemic, researchers have published clinical results allowing accurate antibody tests to be distinguished from those that are unreliable. Recently published studies have led the FDA to revoke some EUAs and to support others, such as the Abbott assay. The test-retest performance of the Superbio test raises a major concern about the meaning of a positive test: of the 28 IgM-positive results, only 53.5% tested positive; of the 30 IgG-positive samples that were retested, the second test was positive in only 76.6% of cases. When participants who tested positive for IgM only with the Superbio assay were removed from statistical analysis, the percentage of antibody-positive individuals who subsequently tested positive for IgG on the Abbott assay increased to 3/12 (25%).

Considering the results provided by the FDA-approved Abbott assay as true positives implies an overall seroprevalence in the study group of 2%. This value indicates a seroprevalence among HCWs that is slightly higher than that reported for the local general population.^19^ This pattern of increased SARS-CoV-2 seroprevalence among HCWs has been reported previously.^28^ In a study of SARS-CoV-2 seroprevalence among ED HCWs, Stubblefield et al^30^ reported a seropositivity rate of 7.6%, which is similar to the values reported here. The study found that almost half of the HCWs who were seropositive were asymptomatic, which is similar to the trend observed in our study. These findings could be used to select a cohort of HCWs that would benefit from additional screening.

Although SARS-CoV-2 seroprevalence was higher among ED HCWs at our institution than among the county’s working-age population, this trend was not reflected in the results of RT-PCR testing. Only one participant tested positive for SARS-Cov-2 RNA. Notably, an infection contracted long before study participation would result in positive serology on Day 1, but not necessarily positive results on RT-PCR. This study would benefit from replication at additional sites that draw from larger samples of ED staff.

## LIMITATIONS

Our study has several limitations. When performed in a setting of low prevalence (<5%), serology testing carries a high risk of false positives.^31^ SARS-CoV-2 serology is known to have high cross-reactivity with other common-cold coronaviruses. However, the results of expanded respiratory virus PCR testing showed that none of the SARS-CoV-2 IgM-positive individuals in our study tested positive for any of the four coronaviruses associated with the common cold. Another factor that must be considered when interpreting our results is that, although high test-retest reliability in analyzing serum samples was reported by the manufacturers of the Superbio lateral-flow test, we noted poor test-retest reliability in the ED setting. This finding suggests that variation in the fingerstick test procedure may be a source of variability and decreased sensitivity. Daily variation in serum antibody titers may also have contributed to false-negative results. Finally, high-risk aerosolization procedures (eg, intubation) are performed less frequently in pediatric vs adult healthcare settings, due to the decreased morbidity of COVID-19 in pediatric patients. This could limit the extrapolation of our results to adult settings.

Depending on the method used for analysis, the seroprevalence of SARS-CoV-2 among the pediatric ED HCWs included in this study ranged from 2–10.5%. We observed a seroconversion rate of approximately one new case every two days. Periodically screening HCWs using a rapid antibody screen may help to identify asymptomatic individuals in high-risk settings and thus limit the spread of COVID-19.

Finally, it should be noted that after the study period had ended, the Superbio SARS-CoV-2 IgM/IgG Antibody Fast Detection Kit (Colloidal Gold) was found to have low specificity (83% per the manufacturer and when tested by the CDC). Furthermore, the results of repeat testing with the more-specific Abbot Architect SARS-CoV-2 IgG assay suggest that the number of positive cases reported in this study may be overstated. Despite these limitations, the findings presented above may help those working in the healthcare setting to understand that relying upon devices that lack high levels of specificity may impact the results of tests run on study participants.

## Figures and Tables

**Table 1:** Summary table of subject demographics (N = 143).

Demographics	Percentage
Age	
18–30	41.96%
31–40	30.07%
41–50	18.11%
Over 50	9.79%
Race/ethnicity	
Asian or Asian American	15.38%
Black or African American	1.40%
Hispanic/Latino	15.38%
Multiracial	10.49%
Pacific Islander	2.10%
White or Caucasian	55.24%
Years of experience	
Range	1–40 years
Mean	10.31
Position type	
Full-time	88.81%
Part-time	4.90%
Per diem	6.29%
Position title	
Physician	12.59%
Allied health (PA/NP)	7.69%
Registered nurse	46.85%
ED technician	25.17%
Unit secretary	2.80%
ED administration	4.90%
